# Circulation long non-coding RNAs act as biomarkers for predicting tumorigenesis and metastasis in hepatocellular carcinoma

**DOI:** 10.18632/oncotarget.2934

**Published:** 2015-02-25

**Authors:** Junwei Tang, Runqiu Jiang, Lei Deng, Xudong Zhang, Ke Wang, Beicheng Sun

**Affiliations:** ^1^ Liver Transplantation Center of the First Affiliated Hospital, Nanjing Medical University, Nanjing, Jiangsu Province, P.R.China; ^2^ Collaborative Innovation Center for Cancer Personalized Medicine, Nanjing Medical University, Nanjing, Jiangsu Province, P.R.China

**Keywords:** plasma, lncRNA, microarray, risk score function, ROC curve

## Abstract

**BACKGROUND & AIMS:**

Alpha Fetal Protein (AFP) was one of the traditional biomarker for diagnosis of Hepatocellular carcinoma (HCC) clinically, however, with the low specificity of AFP, the early diagnosis or the metastasis prediction of HCC is inferior. A new, minimally invasive and more specificity biomarker for the diagnosis or metastasis prediction of HCC are necessary.

**METHODS:**

In this study, we applied an lncRNA microarray to screen the potential biomarker for HCC. The multi-stage validation and risk score formula detection was used for validation.

**RESULTS:**

We discovered three lncRNA, RP11–160H22.5, XLOC_014172 and LOC149086, which were up-regulated in HCC comparing with the cancer-free controls with the merged area under curve (AUC) in training set and validation set of 0.999 and 0.896. Furthermore, XLOC_014172 and LOC149086 was confirmed highly increased in metastasis HCC patients with the merged AUC in training set and validation set of 0.900 and 0.934. Besides, most patients presented a decreased level of the three lncRNAs after operation, while the patients with secondary increased level might be associated with tumor hematogenous metastasis.

**CONCLUSIONS:**

RP11–160H22.5, XLOC_014172 and LOC149086 might be the potential biomarker for the tumorigenesis prediction and XLOC_014172 and LOC149086 for metastasis prediction in the future.

## INTRODUCTION

Hepatocellular carcinoma (HCC) is the one of the most common malignant tumor with a high mortality in humans [1–2]. Virus infections including HBV and HCV have been regarded as the main factor of HCC in China [3–4]. The pathogenesis of HCC is concealed, its progress is rapid, its prognosis is poor, and the mortality rate is high with the character of aggressiveness, invasiveness, especially intrahepatically, and frequent recurrence after resection [5–6]. Alpha Fetal Protein (AFP) has long been used for the diagnosis or monitoring the recurrence [7–8]; however, despite the high sensitivity of AFP detection, the specificity of AFP detection have frequently been reported poor in clinical application [9–10]. A new, novel factor with high sensitivity and specificity is necessary for the monitoring of early diagnosis or early metastasis of HCC.

The long non-coding RNAs (lncRNAs) were reported as a biomarker for predicting survival, metastasis, and in the diagnosis of multiple diseases [11–13]. LncRNAs were characterized with the relatively stable style with their secondary structure in body fluids, thus, the detection of lncRNAs in human plasma or urine was possible for researchers [14]. lncRNAs have been previously investigated for their potential role as cancer biomarkers in body fluids [15–16]. The MD-miniRNA, derived from MALAT-1, has been explored, and was available for clinical detection for human prostate cancer [17].

Although numerous studies have investigated small RNAs such as microRNAs (miRNAs) as potential biomarkers for the diagnosis or metastasis prediction for HCC [18–19], the diagnostic utility of circulating lncRNAs in HCC has never been explored. In this study, we are approaching to investigate the potential use of circulating lncRNAs in plasma as biomarkers for HCC. By using both Affymetrix lncRNAs microarray and reverse-transcription quantitative polymerase chain reaction (RT-qPCR) assays to characterize the genome-wide lncRNAs expression profile in plasma from HCC patients before operation and the corresponding plasma after operation by comparing with the cancer-free controls, we sought to identify a panel of plasma lncRNAs that might serve as a novel biomarker for diagnosis of HCC.

## RESULTS

### High throughput microarray detection of plasma lncRNAs

Human LncRNA Array v3.0 (Agilent, CA, USA) was applied to detect the LncRNA derived from plasma of patients with HCC (both pre-operation and post-operation) and the cancer-free cohort in this study. For the microarray detection, we randomly chose the plasma samples obtained from three male HCC patients who were undergoing resection before operation and the corresponding plasma after operation for a month, in addition, three plasma extracted from cancer-free volunteers were regarded as control group. The clinicopathological relevance analysis of total 467 patients was summarized in Table 1. All 217 patients enrolled in this study were clinically and pathologically diagnosed with HCC. There were no significant differences in the distribution of age and sex between the cancer patients and the cancer-free controls.

**Table 1:** Clinicopathological features of surgical hepatocellular carcinoma (HCC) and cancer-free control samples

	HCC	CH	Control	*P* value
**N**	217	100	250	
**Age Mean (SE) year**	57.79(0.78)	59.77(0.22)	56.08(0.68)	0.14^a^
**Sex (male/female)**	177/40	83/22	202/48	0.83^b^
**Differentiation grade**				
Well	97			
Moderate	62			
Poorly	58			
**Tumor Size(cm)**				
≤ 5 cm	154			
> 5 cm	63			
**Tumor Number**				
Solitary	101			
Multiple	116			
**Tumor Capsular**				
Incomplete	17			
Complete	200			
**TNM stage(I:II:III)**	101:72:44			
**Metastasis**				
Yes	109			
No	108			
Negative	45			
Missed	10			

a Student *t*-test.

b Chi-square test.

Hierarchical clustering analysis and volcano plot distribution were used to sort the aberrantly expressed lncRNAs between the pre-operation and post-operation group of HCC as well as the HCC group and the control group (Supplementary Figure 2A, 2B). A total of 540 lncRNA transcripts were specifically de-regulated (464 lncRNA transcripts up-regulated and 76 lncRNA transcripts down-regulated; each *p* < 0.05) in patients with HCC compared with cancer-free controls. Furthermore, totally 610 lncRNA transcripts were changed with 403 decreased and 207 increased in HCC patients post-operation comparing with the pre-operation status. In order to screen the biomarker predication the tumorigenesis of HCC, we merged the up-regulated lncRNA transcripts in HCC patients with the decreased lncRNA transcripts in HCC patients post-operation and finally obtained 43 lncRNA transcripts (Supplementary Figure 2C). Next, Filtering of all the 43 deregulated transcripts for high signal intensity (≥ 5) and at least 2-fold deregulation yielded 13 lncRNA candidates which were highlighted in Supplementary Figure 2D, All of the 13 candidate lncRNAs were confirmed to be consistently amplified in all individual samples. The detailed microarray data are available in the ArrayExpress database (http://www.ebi.ac.uk/arrayexpress) under accession number E-MTAB-2563.

### Biomarker selection by training set and validation set

We further examined these differentially expressed lncRNAs by RT-qPCR in a training sample set including 20 cases and 20 controls (including the same samples used in microarray assay). In this phase, we retained only the lncRNAs with a mean fold-change >2 and a *P* value < 0.05. As shown in Supplementary Table 1, this phase generated a panel of three lncRNAs (RP11–160H22.5, XLOC_014172 and LOC149086) that were significantly up-regulated in HCC samples.

**Figure 1:**
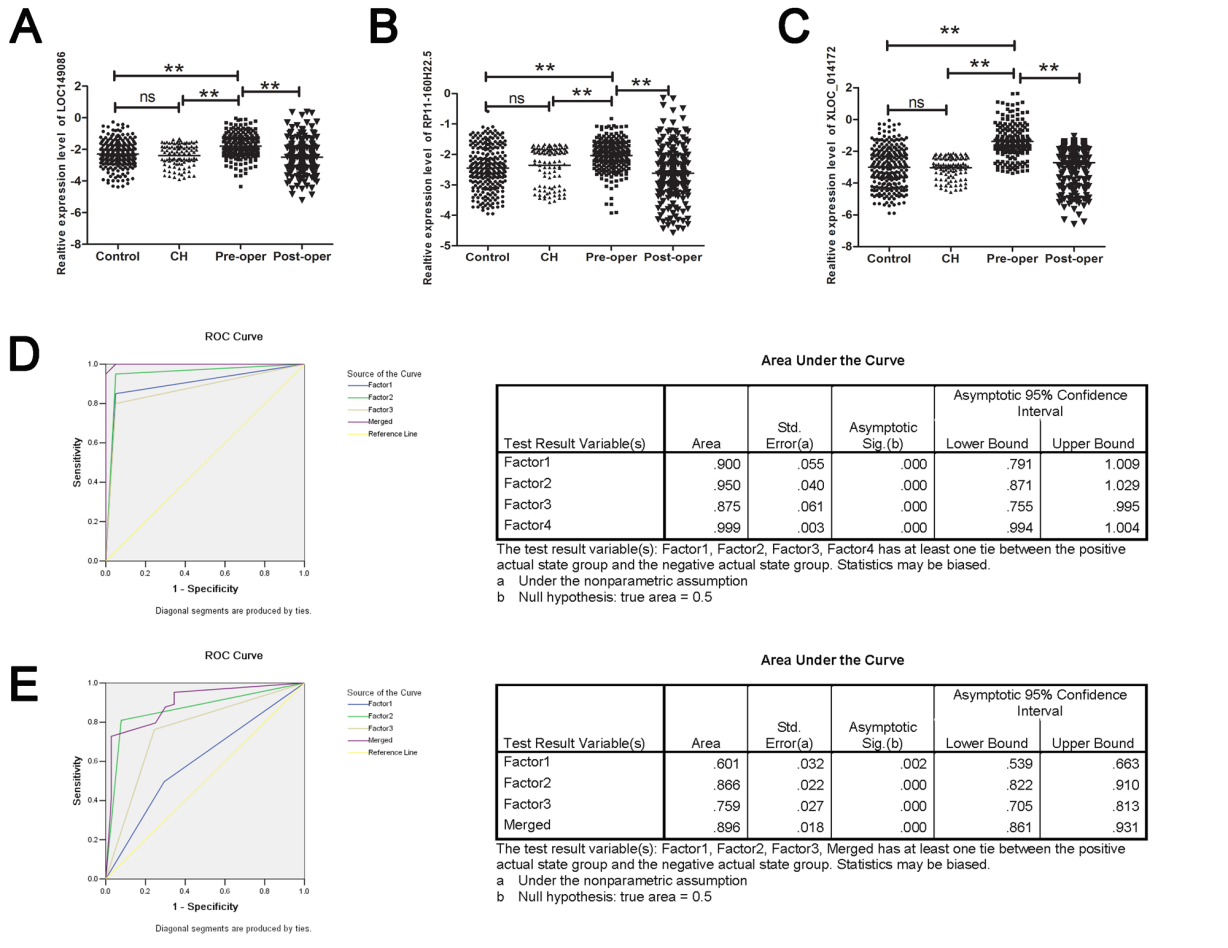
RT-qPCR and ROC curve analysis for predicting the three lncRNAs as a HCC diagnosis biomarker **(A–C)** Total 217 paired plasma from HCC patients 100 CH patients and 250 cancer-free controls were used in RT-qPCR analysis. Data was log-transformed and was presented as mean ± SD. Data was analyzed with student *t* test. ** indicated *p* < 0.01. **(D)** ROC curve analysis was conducted for discrimination between HCC cases and controls by the three-lncRNA profile. ROC curve analysis was performed for the three-lncRNA signature to separate 20 HCC cases from 20 controls in the training set with the AUC presented in the right D. **(E)** ROC curve for the three-lncRNA signature to separate 147 HCC cases from 180 controls in the validation set with the AUC presented in the right. Factor1, 2, 3 and merged represented the RP11–160H22.5, XLOC_014172, LOC149086 and the combination of the three lncRNA.

To validate the accuracy and specificity of these three lncRNA as a HCC potential signature, we also examined their expression levels through a larger individual samples (147 cases and 180 controls). As shown in Supplementary Table 1, the expression of three lncRNAs in plasma of HCC were all significantly higher than those in controls, which was consist with the results in training set. We next merged the total samples to analyze the expression level of three lncRNAs in HCC patients (both pre-operation and post-operation) and the control group. We found that all of the three candidate lncRNAs indicated aberrant increased levels in HCC patients comparing with the control group and the chronic hepatitis (CH) group. Furthermore, a remarkable decreased level was also obtained in the three lncRNAs in the plasma of HCC patients after the hepatectomy operation while the up-regulation of lncRNAs indicated no difference between the CH group and the control group (Figure 1A–1C). Throughout the multiphase testing and analysis, a profile of three lncRNAs might be considered to be the potential signature for the tumorigenesis of HCC.

To assess the diagnostic value of the three lncRNAs profiling system, we used a risk score formula to calculate the risk score function for cases and control samples. First, the risk score of each plasma sample in the training set was calculated, as the basis of their risk scores and a set cut off, plasma samples were then divided into a high-risk group, representing the possible HCC group, and a low-risk group, representing the predicted controls. At the optimal cutoff value (Value = 7.449) with the value of sensitivity + specificity considered to be maximal, the diagnostic sensitivity and specificity of the three lncRNAs signature for the occurrence of HCC were 85% and 95%, and the positive predictive value and negative predictive value was 100% and 95% in the training set, respectively. Similarly, when the same cutoff value was applied to calculate the risk score of samples in the larger validation sets, the diagnostic sensitivity and specificity were 82%, 73%, respectively (Table 2).

**Table 2:** Risk score analysis of in hepatocellular carcinoma HCC and cancer-free control plasma samples

Score	0–7.449	7.449–14.89	PPV^a^	NPV^b^
**Training set**			1.00	0.95
HCC	1	19		
Control	20	0		
**Validation set**			0.82	0.73
HCC	45	135		
Control	117	30		

a PPV, positive predictive value.

b NPV, negative predictive value.

The ROC curves analysis was then conducted to assess the diagnostic sensitivity and specificity of the three-lncRNAs signature for HCC by using these risk score functions (RSFs). Single lncRNA and merged factors were analyzed respectively. As we presented in Figure 1D, the areas under the curve (AUC) of RP11–160H22.5, XLOC_014172, LOC149086 and the merged factor were 0.900, 0.950. 0.875, 0.999, respectively in training set, and in the validation set the AUC of which were 0.601, 0.866, 0.759, 0.896, respectively (Figure 1E).

### Double-blind test for validating the diagnostic capability

We tested another 100 plasma samples (50 HCC patients and 50 cancer-free controls) in a double-blind fashion to validate the accuracy of the three plasma lncRNA biomarker for the detection of HCC diagnosis. After analyzing the expression levels of the three lncRNAs in these samples and classifying them on the basis of previously built diagnostic model (risk score formula), a clear separation of HCC cases from controls was observed, with the accuracy rate of the three-lncRNAs profile as a HCC biomarker being 90.0%.

### Detailed clinicopathological relevance analysis in HCC patients

**Figure 2:**
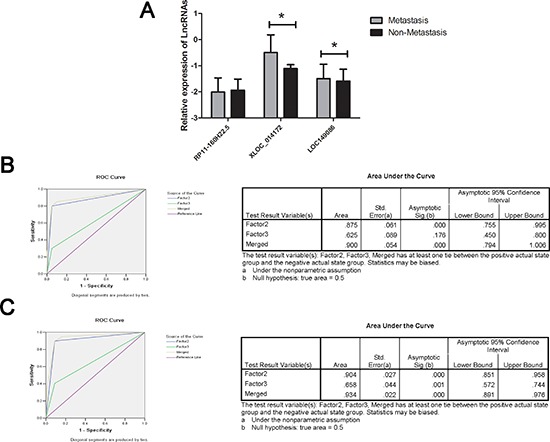
RT-qPCR and ROC curve analysis for predicting lncRNAs as a HCC metastasis biomarker **(A)** Total 109 plasma from HCC patients with metastasis and 108 non-metastasis HCC patients were used in RT-qPCR analysis. Data was presented as mean ± SD and was analysis with student *t* test. * indicated *p* < 0.05. **(B)** ROC curve analysis was conducted for discrimination between metastasis group and non-metastasis group by the two-lncRNA profile. ROC curve analysis was performed for the two-lncRNA signature to separate 20 pairs in the training set with the AUC presented in the right **(C)** ROC curve for the two-lncRNA signature to separate 79 HCC cases from 78 controls in the validation set with the AUC presented in the right. Factor 2, 3 and merged represented the XLOC_014172, LOC149086 and the combination of the two lncRNA.

The progression and the prognosis of HCC was highly associated with the clinicopathological features including the tunor size especially the small hepatocellular carcinoma (SHCC), differentiation, metastasis and the HBV or HCV infection [20]. Thus, based on the data obtained above, we analyzed in detail the expression level of the three lncRNAs in there subgroups. Unfortunately,we found that there was no significant difference in the subgroups divided according to the tumor size (5 cm as the cutoff), the differentiation(high, medium or low) or the virus infection(HBV, HCV or negative) (Supplementary Figure 4A–4C). Interestingly, two of the three lncRNA, XLOC_014172, LOC149086, showed a higher expression in plasma sample in HCC patients with metastasis (Figure 2A).

### XLOC_014172 and LOC149086 acting as the metastasizing indicator for HCC

We randomly chose 20 HCC patients with metastasis and 20 non-metastasis HCC patients as the training set, another 79/78 patients as validation set. The same RSF analysis was applied for investigating efficiency of diagnosis. The up-regulation level of the two lncRNAs was confirmed in both the training set and the validation set (Supplementary Table 2). At the optimal cutoff value (Value = 3.214), the diagnostic sensitivity and specificity of the two lncRNAs signature for the metastasis of HCC were 90% and 90%, and the positive predictive value and negative predictive value was 94% and 86% in the training set. Similarly, when the same cutoff value was applied to calculate the risk score of samples in the larger validation sets, the diagnostic sensitivity and specificity were 91%, 90%, respectively (Table 3). In addition, the areas under the curve (AUC) of XLOC_014172, LOC149086 and the merged factor were 0.875, 0.625. 0.900, respectively in training set, and in the validation set the AUC of which were 0.904, 0.658, 0.934, respectively, indicating that the two factors might be a novel predictor for the metastasis of HCC (Figure 2B, 2C).

**Table 3:** Risk score analysis of in hepatocellular carcinoma (HCC) metastasis and non-metastasis patients' plasma samples

Score	0–3.214	3.214–6.428	PPV^a^	NPV^b^
**Training set**			0.94	0.86
Non-Metastasis	19	1		
Metastasis	3	17		
**Validation set**			0.91	0.90
Non-metastasis	71	7		
Metastasis	8	71		

a PPV, positive predictive value.

b NPV, negative predictive value.

### Origin predication of the endogenous RP11–160H22.5, XLOC_014172 and LOC149086

Previous researches have reported that the aberrant expression level of non-coding RNA such as miRNA in tumor patients could be the result of the occurrence of the tumor. In order to explain the origin of the lncRNAs detected in our study, we compared the expression level of the three lncRNAs in the pre-operation group and the post-operation group to investigate whether the up-regulation level of the three lncRNAs was induced by the existence of tumor. Total 217 patients with HCC was detected, a remarkable decreased level of the three lncRNA was observed after the tumor resection for a month with *P* < 1 × 10^−10^(Supplementary Table 3).

**Figure 3:**
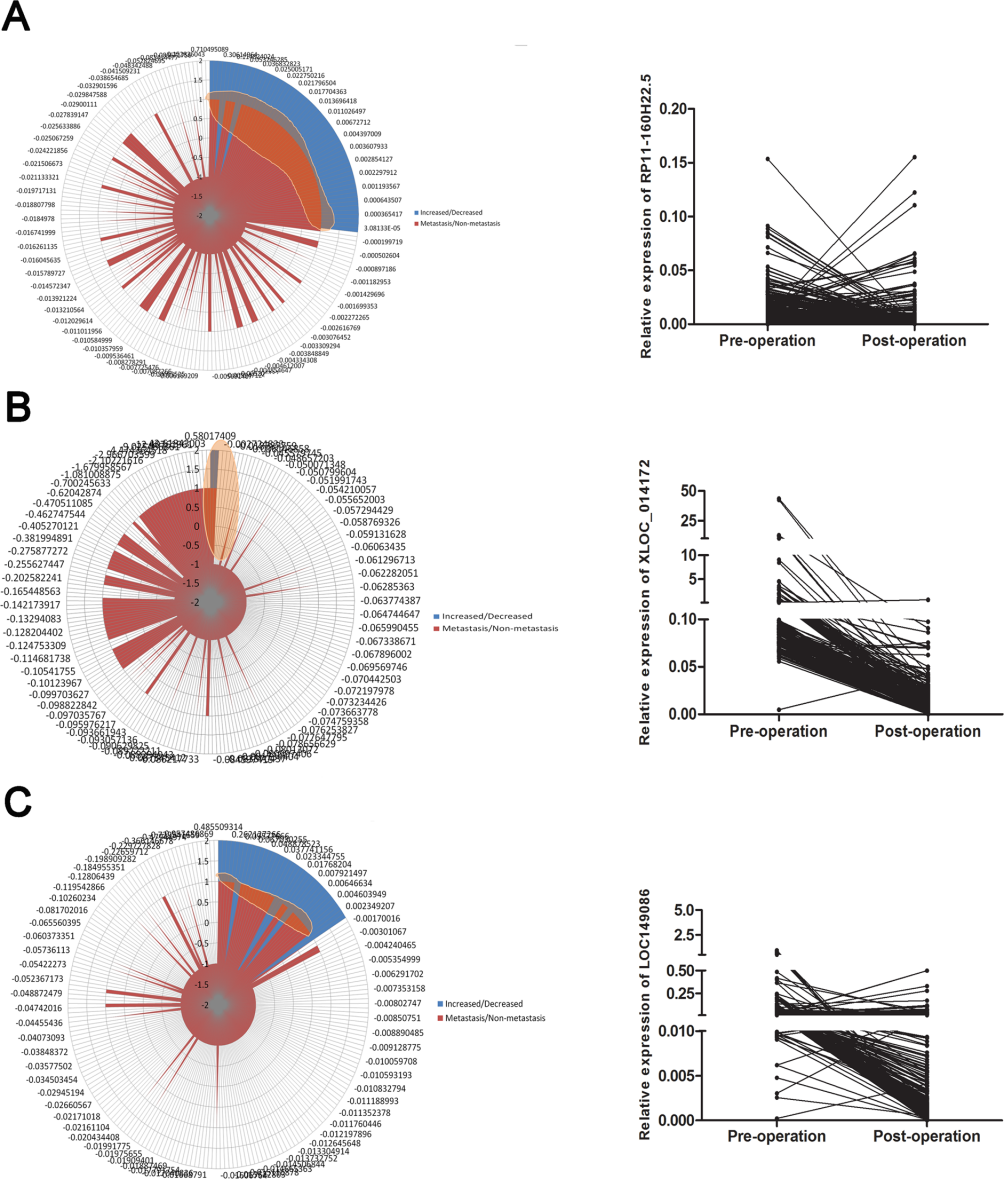
Secondary increased level of lncRNAs after operation was associated with metastasis **(A–C)** (left panel) Radar map method was used for analyzing the distribution of the patients with secondary increased level of lncRNAs after operation, the highlighted areas indicated the patients with the common features of metastasis and secondary increased lncRNA. A–C (right panel) The expression level of three lncRNA before operation and after operation. The major patients presented a tendency of down-regulation while a portion of which indicated an abnormal increased level.

A deeper analysis was performed which indicated that most of the patients presented a decreased level of the three levels after operation; however, some patients indicated an increased level after operation as was showed in Figure 3A–3C in the right panel. We were approaching to explain the abnormal up-regulation level of lncRNAs after operation expression by combining the expression level with clinical information. According to the results reported, the abnormal increased factor originate from the primary tumor was highly associated with the metastasis. We further calculated the correlation between the de-regulation and metastasis of the three lncRNAs. The results indicated that mainly of the patients with increased level of lncRNAs after operation share the feature of metastasis (Supplementary Table 4). Besides, as presented in Figure 3A–3C in the left panel, the results of radar map method indicated that the majority abnormal elevatation was highly matched with the metastasis of HCC.

### Stability detection of endogenous RP11–160H22.5, XLOC_014172 and LOC149086 in human plasma

We first amplified the three lncRNAs in three healthy controls, and detected the product of amplification by agarose electrophoresis. The bands presented in Supplementary Figure 5A and 5B indicated that all of the three lncRNAs were detectable in human plasma. We further incubated human plasma obtained from three healthy controls at room temperature for 12 h, 24 h or subjecting it to up to 3 cycles of freezing and thawing. All of the process had minimal effects on the concentrations of the three lncRNAs, demonstrating that these miRNAs are sufficiently stable in human plasma.

## DISCUSSION

The exploration of biomarker for HCC have been undertaking by numerous researchers for decades [21]. Various biomarkers either extracted from tumor tissues or cell-free plasma has been reported predicting the tumorigenesis, metastasis, or prognosis of HCC patients [22–23]. Among the multitudinous biomarkers, AFP was the most frequently used factor for the diagnosis of HCC clinically [24]. Besides, The development of high throughput microarray and secondary generation sequencing have discovered some new biomarkers for HCC such as a miRNA panel (miR-122, miR-192, miR-21, miR-223, miR-26a, miR-27a and miR-801) which have been reported to be considerable clinical value in diagnosing early-stage HCC and to identify novel therapeutic targets [18]. Not only the miRNA, but also the lncRNA have been annotated acting as a biomarker in predicting the feature of tumor [25–26]. For example, genomic mapping studies have identified lncRNA PCA3 as a prostate cancer specific gene [27]; however, such clinical studies on the circulation lncRNA were never performed in HCC.

In our study, we performed a case-control study through the high throughput lncRNA microarray, and discovered three novel lncRNA, RP11–160H22.5, XLOC_014172 and LOC149086, acting as the biomarker for predicting the occurrence of HCC. The risk score analysis including a multistage validation was employed to evaluate the association between HCC and the lncRNAs expression levels. However, due to the intervention of HBV in the pathogenesis of HCC, to distinguish the lncRNA signature from HBV infection, we measured the levels of these lncRNA in plasma samples from patients with CH free of HCC. The results indicated no correlation between the HBV infection and the aberrant expression of lncRNAs. Next, we deeply analyzed the correlation between the expression level of lncRNAs and the clinical feather of HCC including the tumor size, differentiation, virus infection and metastasis. XLOC_014172 and LOC149086 were identified as the predictor for detecting the metastasis of HCC by using the same risk score analysis. Based on the results above, we tried to explain the origin of lncRNAs we detected in plasma by comparing the expression level of lncRNAs in pre-operation group and the post-operation group. Remarkable decreasing level was obtained after operation while a portion of patients indicated an increased level. To investigate the inner reason, we combined the de-regulated level of lncRNAs with the metastasis of HCC patients according to the reported which indicated the abnormal up-regulation was associated with tumor invasion or the hematogenous dissemination of tumor cells [28–29]. We found that all the patients with the abnormal up-regulation after operation presented with the metastasis clinically which indicating that the secondary elevated level lncRNAs post-operation highly associated with the tumor metastasis. Besides, Deep-sequencing based characterization of exosomal RNA from human plasma revealed that at more than 3% of total exosomal RNA is represented by lncRNAs [30–31]. The exosome might be the vector of the lncRNAs in the hematogenous dissemination. The mechanisms accounting for the stability of plasma lncRNAs are not well-understood; they may be protected by exosome encapsulation such as plasma microRNAs [32].

In conclusion, we identified three lncRNAs, RP11–160H22.5, XLOC_014172 and LOC149086, as the potential biomarker for the tumorigenesis prediction and XLOC_014172 and LOC149086 for metastasis prediction in this study. This is only the preliminary study in our work, and is limited by the small sample size. A deeper potential function of the three lncRNAs in regulating the pathogenesis of HCC is necessary for us to explore in the future.

## MATERIALS AND METHODS

### Study design

The study totally enrolled 217 patients who had been pathological diagnosed as HCC at the time been admitted to the department of hepatobiliary surgery in the First Affiliated Hospital of Nanjing Medical University between 2011 and 2013. Informed consent for blood analysis was obtained prior to surgery; another 100 patients with HBV (+) chronic hepatitis free of HCC was included. The study was approved by the Institutional Ethics Committee of the First Affiliated Hospital of Nanjing Medical University. All research was performed in compliance with government policies and the Helsinki Declaration. Experiments were undertaken with the understanding and written consent of each subject.

A multiphase case-control study was designed to identify a plasma lncRNA profile as a signature for HCC (Supplementary Figure 1). In the screening stage, plasma extracted from 20 HCC patients (both the pre-operation and post-operation) and 20 matched controls were subjected for Human LncRNA Array v3.0 (Arraystar, Agilent, USA) to identify the lncRNAs that were differentially expressed between HCC cases and normal controls as well as the aberrant expressed lncRNAs between the pre-operation group and the post-operation group. Thereafter, we performed individual RT-qPCR in the training set phase to further filter signals of the screened lncRNAs. Subsequently, we perfected the number of plasma lncRNAs included as the HCC signature by a validation set phase to confirm the results computed by the front stage in 147 cases and 180 controls. We also randomly selected another 100 samples in a double-blind analysis (the investigators performing the molecular analysis on the blood samples were blinded to the patients' clinical diagnosis) to validate the diagnostic capability of the candidate lncRNAs. The detailed phases of the study for biomarker selection of metastasis was presented in Supplementary Figure 3 and was described in Supplementary Materials.

### RNA extraction and RT-qPCR

Blood samples were collected from each donor were placed in the EDTA-anticoagulant tube. The plasma were separated by centrifugation at 800 g for 10 min at room temperature, followed by a 15-min high-speed centrifugation at 10 000 g at room temperature to completely remove cell debris. The supernatant plasma was recovered and stored at −80°C until analysis. We extracted total RNA from 300 μL plasma by Trizol reagent according to the protocol of manufactory (Invitrogen, CA, USA) as described previously [33]. We carried out RT-qPCR assay with a commercial kit (TAKARA, Japan) as reported [34].

### Microarray analysis of lncRNAs

Total RNA from each sample was quantified by the NanoDrop ND-1000 (Thermo, CA, USA). For microarray analysis, Agilent Array platform was employed. The sample preparation and microarray hybridization were performed based on the manufacturer's standard protocols with minor modifications. Then, each sample was amplified and transcribed into fluorescent cRNA along the entire length of the transcripts without 3′ bias utilizing a random priming method. The labeled cRNAs were hybridized onto the Human LncRNA Array v3.0 (8 × 60K, Arraystar). After having washed the slides, the arrays were scanned by the Agilent Scanner G2505C (Agilent, CA, USA).

### Statistical analysis

Chi-square tests and the student's *t* test analysis of variance were used to evaluate statistical differences in demographic and clinical characteristics. The paired student-test was used to compare differences in plasma lncRNA expression between pre-operation and post-operation. Risk score analysis was performed to investigate the effectiveness of the three-plasma lncRNA signature for HCC predicting as well as metastasis predication analysis as described [35], for detailed methodology, see Supplementary Materials. Frequency tables and ROC curves were then used to evaluate the diagnostic effects of the profiling and to find the appropriate cutoff point, and to validate the procedure and cutoffs in the next validation sample set. Statistical analysis was performed using STATA 10.0, and presented with GraphPad Prism 5.0 software. Results were considered statistically significant at *P* < 0.05.

## SUPPLEMENTARY MATERIALS AND METHODS



## References

[1] Li WQ, Park Y, McGlynn KA, Hollenbeck AR, Taylor PR, Goldstein AM, Freedman ND (2014). Index-based dietary patterns and risk of incident hepatocellular carcinoma and mortality from chronic liver disease in a prospective study. Hepatology.

[2] El-Serag HB (2011). Hepatocellular carcinoma. N Engl J Med.

[3] Yang P, Li QJ, Feng Y, Zhang Y, Markowitz GJ, Ning S, Deng Y, Zhao J, Jiang S, Yuan Y, Wang HY, Cheng SQ, Xie D, Wang XF (2012). TGF-beta-miR-34a-CCL signaling-induced Treg cell recruitment promotes venous metastases of HBV-positive hepatocellular carcinoma. Cancer Cell.

[4] Willimsky G, Schmidt K, Loddenkemper C, Gellermann J, Blankenstein T (2013). Virus-induced hepatocellular carcinomas cause antigen-specific local tolerance. J Clin Invest.

[5] Sherman M (2008). Recurrence of hepatocellular carcinoma. N Engl J Med.

[6] Yamashita T, Wang XW (2013). Cancer stem cells in the development of liver cancer. J Clin Invest.

[7] Shen Q, Fan J, Yang XR, Tan Y, Zhao W, Xu Y, Wang N, Niu Y, Wu Z, Zhou J, Qiu SJ, Shi YH, Yu B, Tang N, Chu W, Wang M (2012). Serum DKK1 as a protein biomarker for the diagnosis of hepatocellular carcinoma: a large-scale, multicentre study. Lancet Oncol.

[8] Arrieta O, Cacho B, Morales-Espinosa D, Ruelas-Villavicencio A, Flores-Estrada D, Hernandez-Pedro N (2007). The progressive elevation of alpha fetoprotein for the diagnosis of hepatocellular carcinoma in patients with liver cirrhosis. BMC Cancer.

[9] Di Carlo I, Mannino M, Toro A, Ardiri A, Galia A, Cappello G, Bertino G (2012). Persistent increase in alpha-fetoprotein level in a patient without underlying liver disease who underwent curative resection of hepatocellular carcinoma. A case report and review of the literature. World J Surg Oncol.

[10] Hsieh CB, Chen TW, Chu CM, Chu HC, Yu CP, Chung KP (2010). Is inconsistency of alpha-fetoprotein level a good prognosticator for hepatocellular carcinoma recurrence?. World J Gastroenterol.

[11] Dorn GW (2014). LIPCAR: A Mitochondrial lnc in the Noncoding RNA Chain?. Circ Res.

[12] Kumarswamy R, Bauters C, Volkmann I, Maury F, Fetisch J, Holzmann A, Lemesle G, de Groote P, Pinet F, Thum T (2014). Circulating Long Noncoding, RNA, LIPCAR, Predicts Survival in Patients With Heart Failure. Circ Res.

[13] Li J, Chen Z, Tian L, Zhou C, He MY, Gao Y, Wang S, Zhou F, Shi S, Feng X, Sun N, Liu Z, Skogerboe G, Dong J, Yao R, Zhao Y (2014). LncRNA profile study reveals a three-lncRNA signature associated with the survival of patients with oesophageal squamous cell carcinoma. Gut.

[14] Reis EM, Verjovski-Almeida S (2012). Perspectives of Long Non-Coding RNAs in Cancer Diagnostics. Front Genet.

[15] Arita T, Ichikawa D, Konishi H, Komatsu S, Shiozaki A, Shoda K, Kawaguchi T, Hirajima S, Nagata H, Kubota T, Fujiwara H, Okamoto K, Otsuji E (2013). Circulating long non-coding RNAs in plasma of patients with gastric cancer. Anticancer Res.

[16] Xie H, Ma H, Zhou D (2013). Plasma HULC as a promising novel biomarker for the detection of hepatocellular carcinoma. Biomed Res Int.

[17] Ren S, Wang F, Shen J, Sun Y, Xu W, Lu J, Wei M, Xu C, Wu C, Zhang Z, Gao X, Liu Z, Hou J, Huang J (2013). Long non-coding RNA metastasis associated in lung adenocarcinoma transcript 1 derived miniRNA as a novel plasma-based biomarker for diagnosing prostate cancer. Eur J Cancer.

[18] Zhou J, Yu L, Gao X, Hu J, Wang J, Dai Z, Wang JF, Zhang Z, Lu S, Huang X, Wang Z, Qiu S, Wang X, Yang G, Sun H, Tang Z (2011). Plasma microRNA panel to diagnose hepatitis B virus-related hepatocellular carcinoma. J Clin Oncol.

[19] Chang RM, Yang H, Fang F, Xu JF, Yang LY (2014). MicroRNA-331–3p promotes proliferation and metastasis of hepatocellular carcinoma by targeting PH domain and leucine-rich repeat protein phosphatase. Hepatology.

[20] Vitale A, Morales RR, Zanus G, Farinati F, Burra P, Angeli P, Frigo AC, Del Poggio P, Rapaccini G, Di Nolfo MA, Benvegnu L, Zoli M, Borzio F, Giannini EG, Caturelli E, Chiaramonte M (2011). Barcelona Clinic Liver Cancer staging and transplant survival benefit for patients with hepatocellular carcinoma: a multicentre, cohort study. Lancet Oncol.

[21] Shang S, Plymoth A, Ge S, Feng Z, Rosen HR, Sangrajrang S, Hainaut P, Marrero JA, Beretta L (2012). Identification of osteopontin as a novel marker for early hepatocellular carcinoma. Hepatology.

[22] Matsubara T, Kanto T, Kuroda S, Yoshio S, Higashitani K, Kakita N, Miyazaki M, Sakakibara M, Hiramatsu N, Kasahara A, Tomimaru Y, Tomokuni A, Nagano H, Hayashi N, Takehara T (2013). TIE2-expressing monocytes as a diagnostic marker for hepatocellular carcinoma correlates with angiogenesis. Hepatology.

[23] Sun YF, Xu Y, Yang XR, Guo W, Zhang X, Qiu SJ, Shi RY, Hu B, Zhou J, Fan J (2013). Circulating stem cell-like epithelial cell adhesion molecule-positive tumor cells indicate poor prognosis of hepatocellular carcinoma after curative resection. Hepatology.

[24] Yamashita T, Kitao A, Matsui O, Hayashi T, Nio K, Kondo M, Ohno N, Miyati T, Okada H, Mizukoshi E, Honda M, Nakanuma Y, Takamura H, Ohta T, Nakamoto Y, Yamamoto M (2014). Gd-EOB-DTPA-enhanced magnetic resonance imaging and alpha-fetoprotein predict prognosis of early-stage hepatocellular carcinoma. Hepatology.

[25] Bolton EM, Tuzova AV, Walsh AL, Lynch T, Perry AS (2014). Noncoding RNAs in prostate cancer: the long and the short of it. Clin Cancer Res.

[26] Quagliata L, Matter MS, Piscuoglio S, Arabi L, Ruiz C, Procino A, Kovac M, Moretti F, Makowska Z, Boldanova T, Andersen JB, Hammerle M, Tornillo L, Heim MH, Diederichs S, Cillo C (2014). Long noncoding RNA HOTTIP/HOXA13 expression is associated with disease progression and predicts outcome in hepatocellular carcinoma patients. Hepatology.

[27] Bussemakers MJ, van Bokhoven A, Verhaegh GW, Smit FP, Karthaus HF, Schalken JA, Debruyne FM, Ru N, Isaacs WB (1999). DD3: a new prostate-specific gene, highly overexpressed in prostate cancer. Cancer Res.

[28] Shantha Kumara HM, Tohme ST, Herath SA, Yan X, Senagore AJ, Nasar A, Kalady MF, Baxter R, Whelan RL (2012). Plasma soluble vascular adhesion molecule-1 levels are persistently elevated during the first month after colorectal cancer resection. Surg Endosc.

[29] Antoniewicz AA, Paziewska A, Mikula M, Goryca K, Dabrowska M, Poletajew S, Borowka A, Ostrowski J (2012). Lack of evidence for increased level of circulating urothelial cells in the peripheral blood after transurethral resection of bladder tumors. Int Urol Nephrol.

[30] Huang X, Yuan T, Tschannen M, Sun Z, Jacob H, Du M, Liang M, Dittmar RL, Liu Y, Kohli M, Thibodeau SN, Boardman L, Wang L (2013). Characterization of human plasma-derived exosomal RNAs by deep sequencing. BMC Genomics.

[31] Shah S, Wittmann S, Kilchert C, Vasiljeva L (2014). lncRNA recruits RNAi and the exosome to dynamically regulate pho1 expression in response to phosphate levels in fission yeast. Genes Dev.

[32] Arroyo JD, Chevillet JR, Kroh EM, Ruf IK, Pritchard CC, Gibson DF, Mitchell PS, Bennett CF, Pogosova-Agadjanyan EL, Stirewalt DL, Tait JF, Tewari M (2011). Argonaute2 complexes carry a population of circulating microRNAs independent of vesicles in human plasma. Proc Natl Acad Sci U S A.

[33] Tang W, Qin J, Tang J, Zhang H, Zhou Z, Li B, Geng Q, Wu W, Xia Y, Xu X (2013). Aberrant reduction of MiR-141 increased CD47/CUL3 in Hirschsprung's disease. Cell Physiol Biochem.

[34] Tang W, Tang J, Qin J, Geng Q, Zhou Z, Li B, Zhang J, Chen H, Xia Y, Wang X (2013). Involvement of down-regulated E2F3 in Hirschsprung's disease. J Pediatr Surg.

[35] Liu R, Chen X, Du Y, Yao W, Shen L, Wang C, Hu Z, Zhuang R, Ning G, Zhang C, Yuan Y, Li Z, Zen K, Ba Y, Zhang CY (2012). Serum microRNA expression profile as a biomarker in the diagnosis and prognosis of pancreatic cancer. Clin Chem.

