# Supplementary material for: Circulation long non-coding RNAs act as biomarkers for predicting tumorigenesis and metastasis in hepatocellular carcinoma
Source: Oncotarget. 2015 Feb 25;6(6):4505–15. doi: 10.18632/oncotarget.2934 (PMC4414207; doi:10.18632/oncotarget.2934)
Supplement: Supplementary file 1 [file oncotarget-06-4505-s001.pdf]

SUPPLEMENTARY MATERIALS AND METHODS

LNCRNA EXPRESSION MICROARRAY DETECTION AND DATA ANALYSIS

Agilent Feature Extraction software (version 11.0.1.1) was used to analyse acquired array images. Quantile normalization and subsequent data processing were performed using the GeneSpring GX v12.0 software package (Agilent Technologies). After quantile normalization of the raw data, LncRNAs that at least 3 out of 9 samples have flags in present or marginal (“All Targets Value”) were chosen for further data analysis. Differentially expressed LncRNAs with statistical significance between the two groups were identified through Volcano Plot filtering. Finally, Hierarchical Clustering was performed to show the distinguishable LncRNAs expression pattern among samples.

Risk score analysis

Risk score analysis was performed to evaluate the associations between the concentrations of the plasma lncRNA expression levels. The upper 95% reference interval of each lncRNA value in controls or non-metastasis group was set as the threshold to code the expression level of the corresponding lncRNA for each sample as 0 and 1 in the training set. A risk score function (RSF) to predict HCC or metastasis group was defined according to a linear combination of the expression level for each lncRNA. For example, the RSF for sample i using information from three lncRNAs was: $rsfi = \Sigma 3j-1Wj.sij$. In the above equation, sij is the risk score for lncRNA j on sample i, and Wj is the weight of the risk score of lncRNA j. The risk score of three lncRNAs was calculated using the weight by the regression coefficient that was derived from the univariate logistic regression analysis of each lncRNAs. Samples were ranked according to their RSF and then divided into a high-risk group, representing the predicted HCC cases or metastasis patients, and a low-risk group, representing the predicted control individuals or non-metastasis patients. Frequency tables and ROC curves were then used to evaluate the diagnostic effects of the profiling and to find the appropriate cutoff point, and to validate the procedure and cutoffs in the next validation sample set.

SUPPLEMENTARY FIGURES AND TABLES

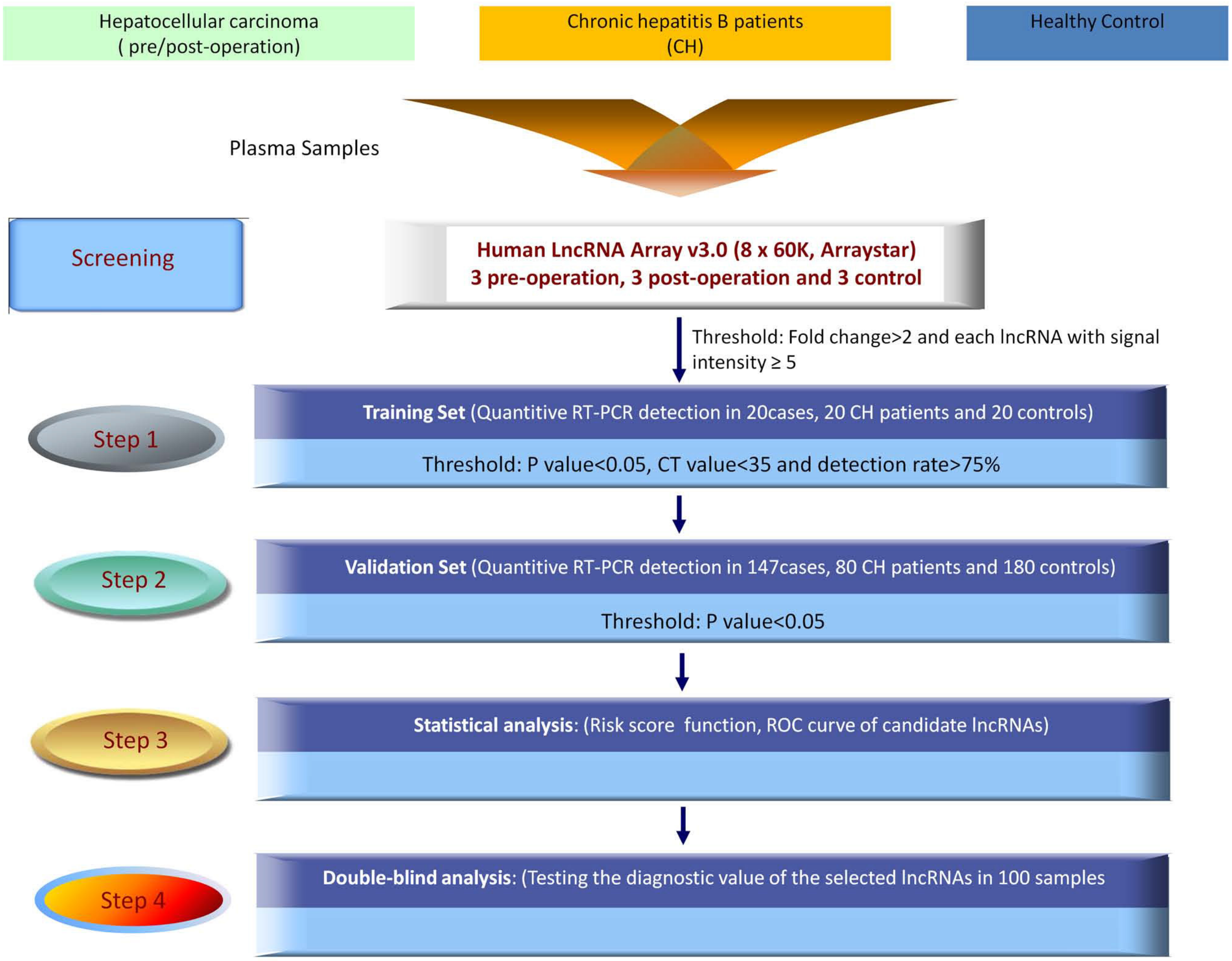

Supplementary Figure S1: Overview of the work design in biomarker screen for the diagnosis of HCC.

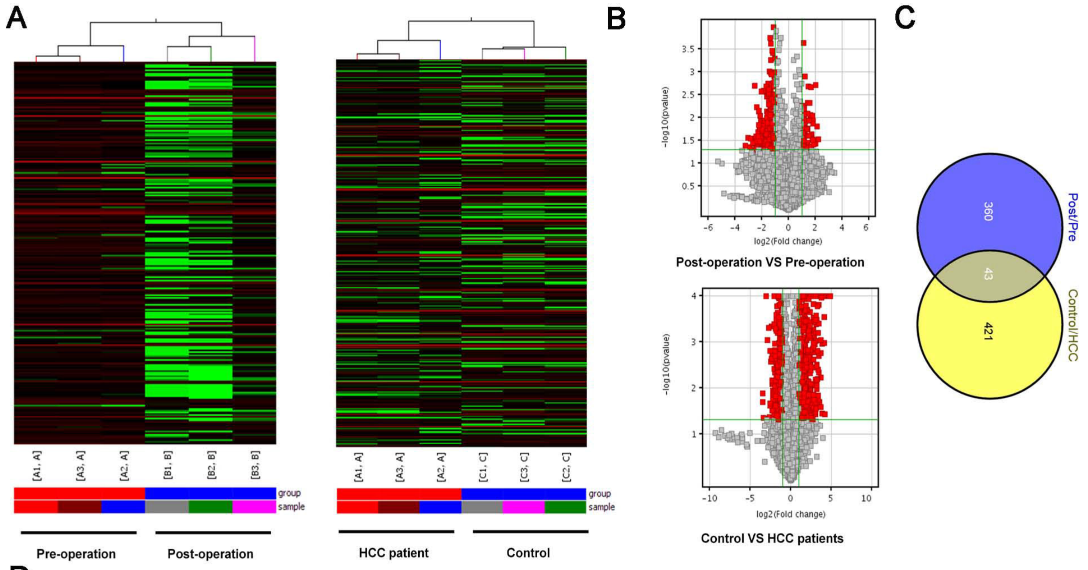

D

Common elements in "Post/Pre" and "Control/HCC":										
		Raw Intensity								
seqname	ProbeName	[A1] (raw)	[A2] (raw)	[A3] (raw)	[B1] (raw)	[B2] (raw)	[B3] (raw)	[C1] (raw)	[C2] (raw)	[C3] (raw)
ENST00000545254	ASHGA5P026619	**106.4653**	**93.01934**	**112.5689**	**7.213182**	**19.15113**	**110.9648**	**25.08888**	**29.54317**	**33.42484**
NR_033905	ASHGA5P019437	**33.54419**	**22.43648**	**33.78111**	**5.058322**	**6.500741**	**22.3771**	**6.425284**	**5.915247**	**10.50696**
ENST00000435996	ASHGA5P053326	**506.2274**	**212.9809**	**493.5091**	**19.39055**	**51.77796**	**352.7547**	**86.27879**	**98.49406**	**95.37294**
TCONS_00001047	ASHGA5P045206	56.576355	21.261923	50.13768	4.9999995	7.6225123	18.58892	9.825793	6.4726553	7.0313096
HMlincRNA1314-	ASHGA5P023657	73.07117	34.859196	68.43905	4.9999995	6.678862	47.018753	16.094	18.96585	7.27399
ENST00000528139	ASHGA5P044802	**68.0651**	**36.29069**	**65.81077**	**5.144844**	**7.853405**	**43.11955**	**13.08881**	**6.488891**	**9.173887**
ENST00000450503	ASHGA5P038133	37.588642	12.818281	26.113565	4.9999995	6.8232074	19.28161	4.9999995	4.9999995	7.226889
uc003jsd.1	ASHGA5P040455	56.197895	20.980192	47.20057	5.227181	7.92679	32.7777	4.9999995	6.364204	8.764888
ENST00000422108	ASHGA5P015632	36.376976	23.034508	35.935856	5.442384	7.5423336	22.12846	4.9999995	7.739988	5.292424
ENST00000561181	ASHGA5P022705	35.93189	11.123838	29.168228	4.9999995	6.500975	24.49001	4.9999995	7.114365	4.9999995
TCONS_00020616	ASHGA5P057673	31.094194	21.716866	33.5137	5.098104	7.1547365	24.013329	7.756229	4.9999995	6.8607106
TCONS_00026233	ASHGA5P036830	62.90411	27.259851	82.44051	5.2578597	8.009199	30.408567	7.717275	4.9999995	10.090679
ENST00000419983	ASHGA5P015406	**118.8226**	**37.92808**	**118.5329**	**5.087275**	**17.32147**	**49.08662**	**5.473594**	**17.43008**	**7.382277**
TCONS_00027298	ASHGA5P036155	35.63867	33.013535	40.53198	5.407441	7.534977	26.332687	4.9999995	5.524394	4.9999995
TCONS_00020424	ASHGA5P041842	**62.37462**	**25.69455**	**61.97341**	**5.019105**	**7.42607**	**44.29219**	**7.480561**	**9.26018**	**6.427513**
uc003anf.2	ASHGA5P018420	**51.5182**	**33.28681**	**41.26122**	**5.038707**	**7.482239**	**36.69275**	**11.59338**	**8.871861**	**6.282064**
TCONS_00029527	ASHGA5P032219	**76.37541**	**29.08278**	**76.52879**	**8.259509**	**12.46773**	**39.33324**	**16.02031**	**15.72527**	**7.654149**
ENST00000544557	ASHGA5P021921	**37.50307**	**29.3238**	**34.81741**	**5.097444**	**7.723309**	**34.22229**	**12.32377**	**7.623465**	**6.551936**
ENST00000413791	ASHGA5P035101	**73.53163**	**36.66718**	**81.30029**	**5.772625**	**9.253573**	**39.00427**	**13.5352**	**12.43956**	**16.25209**
uc001bbe.1	ASHGA5P030061	37.96878	13.395019	31.007874	4.9999995	7.764559	23.230122	8.393821	4.9999995	4.9999995
ENST00000525992	ASHGA5P055170	59.80021	26.052486	83.028244	4.9999995	7.265597	28.117723	7.300884	10.73325	15.48507
NR_047694	ASHGA5P025988	47.92929	41.85647	41.793377	5.1225405	7.071056	28.660076	8.729898	4.9999995	14.05652
NR_038205	ASHGA5P025808	40.126987	41.55738	35.960415	5.0579114	8.286993	28.841452	10.82943	9.843657	4.9999995
ENST00000521139	ASHGA5P052699	58.902077	39.132324	53.509373	6.351606	7.23032	23.44439	4.9999995	4.9999995	7.417493
ENST00000532280	ASHGA5P053657	44.424694	28.646126	42.542652	4.9999995	7.1277194	37.461826	7.7106357	14.52451	4.9999995
ENST00000560760	ASHGA5P030182	24.916319	14.392083	25.181978	4.9999995	6.168501	25.364439	5.8404217	4.9999995	4.9999995
ENST00000453795	ASHGA5P018460	44.10294	23.878788	51.609226	4.9999995	7.8330164	36.83131	8.711324	14.193188	5.388711
ENST00000559600	ASHGA5P022611	34.09443	20.415264	30.724691	4.9999995	5.801441	31.433392	4.9999995	5.973521	8.586274
TCONS_00029855	ASHGA5P055559	101.54649	54.17835	104.6779	4.9999995	8.525135	53.35846	5.658084	6.2729287	4.9999995
TCONS_00025722	ASHGA5P058046	60.7939	15.171118	61.126774	4.9999995	5.1577597	22.378363	5.0315285	4.9999995	6.837971
uc001bsw.2	ASHGA5P058327	**55.80721**	**31.75638**	**50.895**	**5.012855**	**7.862521**	**37.15664**	**13.43567**	**6.269689**	**11.5174**
ENST00000421637	ASHGA5P038049	37.194016	11.58832	36.603825	4.9999995	6.3718905	22.848969	7.2154493	4.9999995	4.9999995
ENST00000566418	ASHGA5P028028	44.97146	28.943048	42.48475	4.9999995	6.2795234	38.4791	7.8044415	9.729777	5.557211
uc003fza.3	ASHGA5P058419	25.01944	28.634766	27.510908	4.9999995	5.7956004	18.684792	4.9999995	9.433593	4.9999995
NR_024594	ASHGA5P037084	158.75319	24.513187	138.7779	4.9999995	6.843428	42.357914	8.838197	14.206752	16.04173
NR_024594	ASHGA5P037084	158.75319	24.513187	138.7779	4.9999995	6.843428	42.357914	8.838197	14.206752	16.04173
NR_024594	ASHGA5P037084	158.75319	24.513187	138.7779	4.9999995	6.843428	42.357914	8.838197	14.206752	16.04173
ENST00000431981	ASHGA5P045701	75.21143	21.697845	72.6581	5.119525	6.17952	25.081114	4.9999995	4.9999995	4.9999995
NR_038429	ASHGA5P017167	71.72874	15.613947	62.682415	4.9999995	6.731358	22.131159	4.9999995	4.9999995	4.9999995
uc002ain.1	ASHGA5P030082	68.36105	42.883743	74.693504	4.9999995	8.834697	22.509003	4.9999995	16.259861	8.317313
ENST00000493521	ASHGA5P018917	103.25588	19.038046	88.98173	5.1491146	6.2427444	18.51409	11.94445	8.239752	4.9999995
chr7:149967450-150001750+	ASHGA5P000496	**166.7526**	**57.5431**	**104.5749**	**5.042436**	**18.17793**	**32.29109**	**16.44231**	**12.56438**	**16.3757**
NR_046358	ASHGA5P014779	**74.97426**	**72.00106**	**67.33324**	**5.013899**	**8.606903**	**64.83798**	**16.8646**	**19.87151**	**12.11475**

Supplementary Figure S2: Schematic representation of the lncRNA microarray assay. Samples were obtained from three patients with HCC before operation and the corresponding plasma after operation as well as three cancer-free controls. **(A)** Hierarchical clustering analysis was applied for analysis the different expressed lncRNA in patients' plasma before and after operation (left panel). A case-control study was also conducted (right panel). **(B)** Volcano plot distribution was used to sort the aberrantly expressed lncRNAs between the pre-operation and post-operation group as well as the HCC group and the control group. **(C)** The up-regulated lncRNA transcripts in HCC patients with the decreased lncRNA transcripts in HCC patients post-operation were merged, and finally obtained 43 lncRNA transcripts. **(D)** All the 43 deregulated transcripts were filtered by high signal intensity (≥ 5) and at least 2-fold deregulation which yielded 13 lncRNA candidates highlighted.

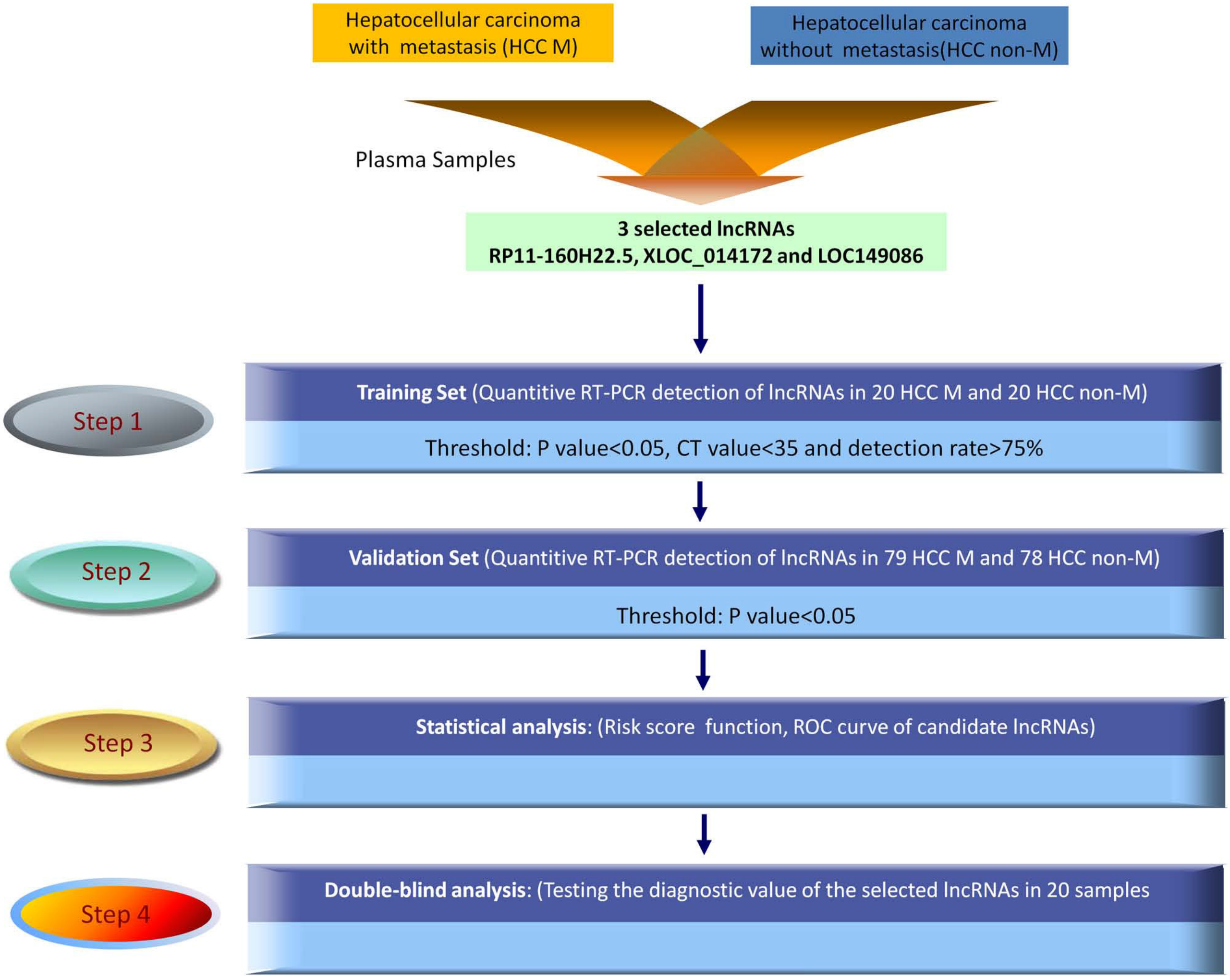

Supplementary Figure S3: Overview of the work design in potential biomarker screening for the metastasis of HCC.

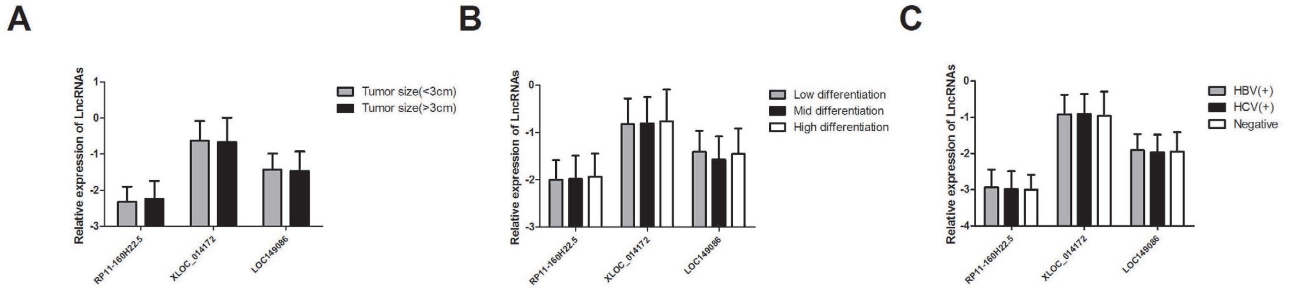

Supplementary Figure S4: Relative expression level of three lncRNAs in subgroup of HCC. **(A)** HCC patients were divided into two groups accoding to the size of the tumor (3 cm as cutoff). **(B)** Patients were groups by the differentiation of tumor according to the pathological diagnosis. **(C)** The sectionalization was based on the virus infection of patients detected clinically including HBV, HCV and no virus infection. Data was presented as mean ± SEM and was analyzed with student *t* test. No significant difference was obtained in subgroup.

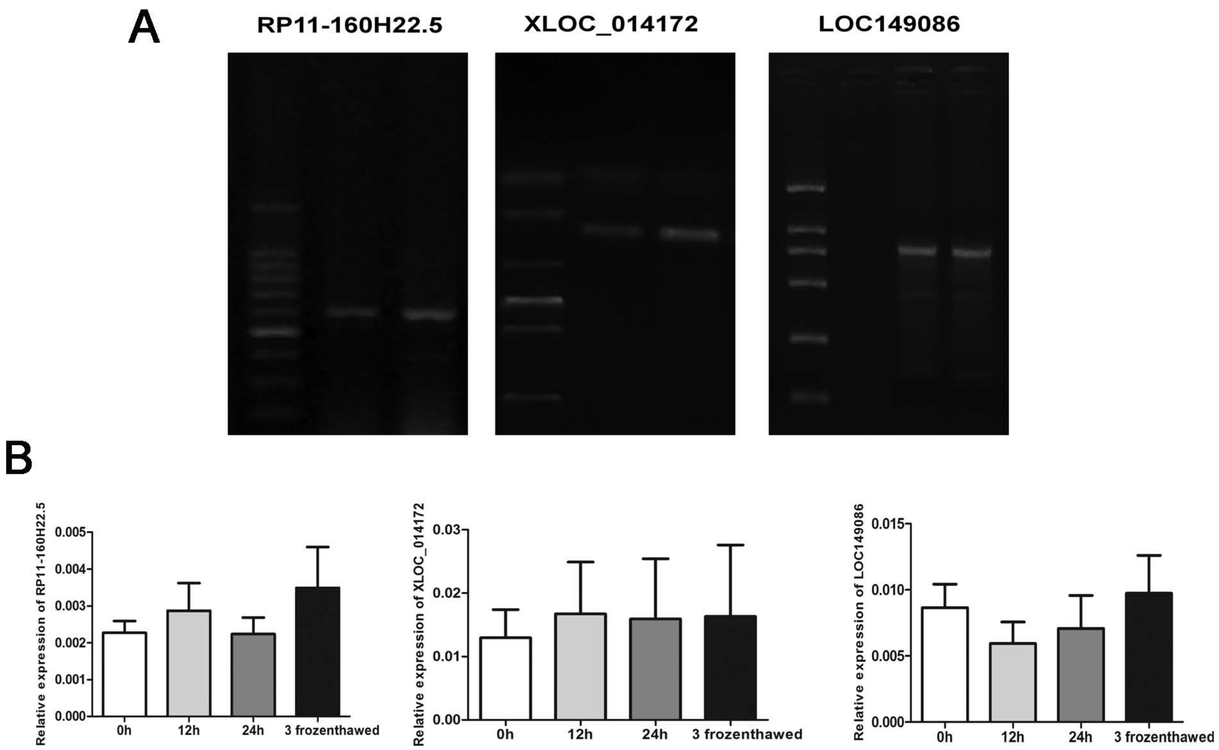

Supplementary Figure S5: Stability detection of endogenous lncRNAs in human plasma. (A) The products of the amplification fragment were detected by agarose electrophoresis. **(B)** RT -qPCR was applied for detecting the expression level of the three lncRNAs. Human plasmas obtained from three healthy controls were incubated at room temperature for 12 h, 24 h or subjecting it to up to 3 cycles of freezing and thawing. Data was presented as mean ± SEM. No significant difference was observed in each group.

Supplementary Table S1: LncRNA expression levels in hepatocellular carcinoma HCC and cancer-free control plasma samples in the training and validation sets

lncRNA	Training set			Validation set		
	HCC	Control	P^b	HCC	Control	P^b
N	20	20		147	180	
RP11–160H22.5	0.014 (0.0089–0.028)	0.0025 (0.0019–0.0045)	3.18×10^{-7}	0.0082 (0.0043–0.019)	0.0033 (0.0011–0.012)	8.90×10^{-8}
XLOC_014172	0.047 (0.1305–0.183)	0.0072 (0.0040–0.020)	4.87×10^{-7}	0.088 (0.076–0.13)	0.0013 (0.00012–0.012)	$< 1 \times 10^{-10}$
LOC149086	0.052 (0.016–1.00)	0.0049 (0.0031–0.010)	4.22×10^{-7}	0.021 (0.013–0.049)	0.0046 (0.0019–0.018)	$< 1 \times 10^{-10}$

[a]Data are expressed as the median (interquartile range).
[b]Wilcoxon rank sum test.

Supplementary Table S2: LncRNA expression levels in hepatocellular carcinoma(HCC) metastasis and non-metastasis patients' plasma samples in the training and validation sets

lncRNA	Training set			Validation set		
	Metastasis	Non-metastasis	P^b	Metastasis	Non-metastasis	P^b
N	20	20		79	78	
XLOC_014172	0.13 (0.10–0.96)	0.082 (0.076–0.089)	0.0006	0.27 (0.12–0.70)	0.017 (0.012–0.037)	$< 1 \times 10^{-10}$
LOC149086	0.04589 (0.028–0.18)	0.020 (0.013–0.035)	0.03	0.019 (0.014–0.039)	0.016 (0.011–0.037)	0.006

[a]Data are expressed as the median (interquartile range).
[b]Wilcoxon rank sum test.

Supplementary Table S3: LncRNA expression levels during perioperation period in hepatocellular carcinoma HCC patients' plasma samples

	Pre-operation[a] ($N = 217$)	Post-operation[a] ($N = 217$)	Fold change	P value[b]
RP11–160H22.5	0.011(0.0061–0.023)	0.0023(0.00083–0.0072)	4.78	$< 1 \times 10^{-10}$
XLOC_014172	0.092(0.076–0.15)	0.011(0.00029–0.022)	8.36	$< 1 \times 10^{-10}$
LOC149086	0.020 (0.014–0.054)	0.0025 (0.00077–0.0099)	8.00	$< 1 \times 10^{-10}$

[a]Data are expressed as the median (interquartile range).
[b]Wilcoxon rank sum test.

Supplementary Table S4: Correlation analysis between lncRNAs expression level variation and the metastasis

		Incerased (N)	Decreased (N)	P value[a]
RP11–160H22.5	Metastasis	57	52	< 0.001
	Non-metastasis	2	106	
XLOC_014172	Metastasis	3	106	0.041
	Non-metastasis	0	108	
LOC149086	Metastasis	31	78	< 0.001
	Non-metastasis	5	103	

[a]Chi-square test.
